# Hysterectomy for placenta accreta spectrum disorder: Impact of institutional surgical volume on patient outcomes

**DOI:** 10.1002/ijgo.70846

**Published:** 2026-02-04

**Authors:** Lacey C. Brennan, Olga Bougie, Jonas Shellenberger, Jessica Pudwell, John Kingdom, Peter S. Thiel, Lisa Allen, Sebastian R. Hobson, Maria P. Velez, Ally Murji

**Affiliations:** ^1^ Department of Obstetrics and Gynecology Peter Lougheed Center Calgary Alberta Canada; ^2^ Department of Obstetrics and Gynecology University of Calgary Calgary Alberta Canada; ^3^ Department of Obstetrics and Gynecology Mount Sinai Hospital Toronto Ontario Canada; ^4^ Department of Obstetrics and Gynecology University of Toronto Toronto Ontario Canada; ^5^ Institute of Clinical Evaluative Sciences (ICES) Kingston Ontario Canada; ^6^ Department of Obstetrics and Gynecology, Queen's University, Kingston Health Sciences Center Kingston General Hospital Kingston Ontario Canada; ^7^ Department of Obstetrics and Gynecology Royal University Hospital, University of Saskatchewan Saskatoon Saskatchewan Canada; ^8^ Department of Obstetrics and Gynecology McGill University Health Center–MUHC Reproductive Center Montreal Quebec Canada; ^9^ Department of Obstetrics and Gynecology, Trillium Health Partners, Institute for Better Health Credit Valley Hospital Mississauga Ontario Canada

**Keywords:** hysterectomy, invasive placentation, maternal morbidity, placenta accreta spectrum, surgical volume

## Abstract

**Objective:**

To evaluate trends in hysterectomy case volume for placenta accreta spectrum (PAS) disorder over time and compare maternal outcomes between high‐ and low‐volume centers. Specifically, we examined whether surgical volume influences severe maternal morbidity (SMM) and other key perioperative outcomes.

**Methods:**

We conducted a population‐based retrospective cohort study of patients aged 18–50 years who underwent hysterectomy for PAS in Ontario, Canada, from January 1, 2003, to January 1, 2022. Cases were identified using provincial administrative health databases. High‐volume centers were defined as institutions performing ≥9 PAS hysterectomies annually, based on probability modeling and receiver operating characteristic analysis. The primary outcome was a validated composite measure of SMM, including massive transfusion, intensive care unit (ICU) admission, surgical complications, readmission, and length of hospital stay.

**Results:**

Among 778 patients, 151 were treated at high‐volume centers. These patients experienced significantly lower rates of SMM compared to those treated at low‐volume centers (45.0% vs. 71.7%; adjusted relative risk [aRR] 0.59, 95% confidence interval [CI]: 0.49–0.72). Risks of massive transfusion (aRR 0.57, 95% CI: 0.47–0.70) and ICU admission (aRR 0.30, 95% CI: 0.15–0.58) were also markedly reduced. Length of hospital stay was shorter at high‐volume centers (1.88 vs. 3.90 days; *P* < 0.0001). Each additional PAS hysterectomy performed in the prior year at a given institution was associated with a 3% reduction in SMM risk (aRR 0.97, 95% CI: 0.96–0.98).

**Conclusion:**

High‐volume centers demonstrate significantly better maternal outcomes for PAS hysterectomy. These findings support centralizing PAS care to improve patient safety and surgical outcomes.

## 1 INTRODUCTION

Cesarean delivery is the most frequently performed inpatient surgery in Canada with 32.5% of all births by this route in 2022–2023.^1^ This rise in cesarean delivery rates has contributed to an increase in complications such as cesarean scar disorder, uterine rupture and placenta accreta spectrum (PAS) disorder in subsequent pregnancies.^2^

PAS is a rare but potentially life‐threatening obstetric complication characterized by abnormal placental invasion into the uterine wall or beyond. PAS ranges in severity from superficial invasion (accreta) to deeper invasion into or through the uterine wall (increta and percreta). The incidence of PAS has been rising, now affecting one in 500 pregnancies in high‐resource countries, largely due to increasing cesarean delivery rates.^3^

Given the substantial maternal morbidity associated with deliveries complicated by a diagnosis of PAS, including catastrophic hemorrhage and an increased risk of preterm delivery, specialized multidisciplinary teams have been developed to improve outcomes. Multiple studies have demonstrated that patients treated by dedicated PAS teams experience less blood loss, require fewer blood transfusions, and experience fewer surgical complications compared to those managed in centers without such specialized care.^4^, ^5^, ^6^, ^7^, ^8^, ^9^

While such teams have proven individually effective, the impact of institutional surgical volume on PAS outcomes at a population‐based level over time remains under‐explored. High surgical volume has been linked to reduced blood loss and fewer surgical complications in all routes of benign hysterectomy,^10^ but the effect on PAS‐hysterectomies is less clear. Given the complexity and rarity of PAS cases, we aimed to examine how institutional surgical volume influences outcomes in PAS‐hysterectomy patients using hospital discharge coding data.

Our study objectives were to examine temporal trends in volume and outcomes with respect to PAS care in Ontario, which is the largest province in Canada. We also sought to compare outcomes between centers with high‐ versus low‐volume of PAS hysterectomies.

## 2 MATERIALS AND METHODS

### 2.1 Study design and data sources

We conducted a population‐based retrospective cohort study of patients who underwent hysterectomy for PAS disorder in Ontario, Canada, between January 1, 2003, and January 1, 2022 using administrative health information databases housed at the Institute for Clinical Evaluative Science (ICES; www.ices.com). ICES is an independent, non‐profit research institute whose legal status under Ontario's health information privacy law allows it to collect and analyze health care and demographic data, without consent, for health system evaluation and improvement. Ethics approval was obtained by Queen's University Health Sciences and Affiliated Teaching Hospitals Research Ethics Board.

We included all pregnant patients between 18 to 50 years of age who underwent a hysterectomy (by any surgical route) with a diagnosis of placenta accreta, increta, or percreta indicated by International Classification of Disease (ICD)‐10 diagnostic codes on their hospital discharge record. We excluded patients with missing or invalid identification numbers, non‐Ontario residents and those with hysterectomy prior to the index admission (within the maximum possible lookback period to April 1, 1993).

The observation period spanned nearly two decades from April 1, 2003, to March 31, 2022. Patient accrual stopped on January 1, 2022 to permit appropriate follow up within the observation window. Patients were followed until the earliest of 42 days post‐discharge, death, loss of Ontario Health Insurance Plan (OHIP) eligibility, or March 31, 2022.

#### 2.1.1 Exposure

The primary exposure of interest was whether hysterectomy was performed at a high‐volume or low‐volume institution. For each index surgery, we calculated the volume of PAS hysterectomies performed at that institution in the year prior. As such, a given institution's volume could change dynamically over time, a method that has been found to be more robust compared to assigning a fixed volume based on an institution's mean case load over the study period.^11^

#### 2.1.2 Statistical analysis to define exposure

To dichotomize institutional volume into high‐ and low‐volume categories, we used various methods in order to choose a clinically meaningful cutoff. We first calculated the probability for the primary outcome across yearly deciles for the distribution of PAS volume. We also graphed fractional polynomial‐transformed continuous volume versus the probability of the primary outcome to evaluate for an inflection point in institutional volume below which there was an impact on maternal morbidity (Figure S1). Finally, we used a logistic regression model of the primary outcome as a function of continuous institutional volume to generate a receiver operating characteristic (ROC) curve, from which we identified the cutoff point with the highest Youden index value (Figure S2). Based on this information, and corroborated with other studies,^12^ we chose a cutoff of ≥9 PAS hysterectomies performed in the prior year to define high‐volume institutions. Low volume centers performed a median of two PAS hysterectomies annually (interquartile range [IQR]). This cutoff demonstrated good sensitivity and positive predictive value of the primary outcome from a low‐volume institution of 86% and 72%, respectively.

#### 2.1.3 Outcomes

The primary outcome was severe maternal morbidity (SMM) within 42 days of hysterectomy, defined using a validated composite measure based on national SMM surveillance criteria.^13^, ^14^, ^15^ In accordance with the operational definitions used by Ray et al. and Aoyama et al., SMM reflects a composite of 21 serious maternal complications and/or life‐saving procedures occurring during pregnancy or up to 42 days postpartum, identified through ICD‐coded diagnoses and interventions.^14^, ^15^ Secondary outcomes included individual components of SMM (including massive transfusions, embolism, shock, disseminated intravascular coagulation [DIC], surgical complications, unplanned reoperations, sepsis, acute renal failure, cardiac complications, death and cerebrovascular events), as well as any blood transfusions, intensive care unit (ICU) admissions, uterine artery embolization (UAE), emergency department visits within 42 days post‐discharge, and length of postoperative hospital admission.

### 2.2 Covariates

We identified patient characteristics a priori as potentially confounding the relationship between institutional volume and the primary outcome. These characteristics included age, comorbidities (using the weighted Johns Hopkins ACG score),^16^ parity, previous abortions, income quintile, rural residence, pre‐existing diabetes, hypertensive disorder of pregnancy, conception method, mode of delivery, gestational age at delivery, obesity, antenatal diagnostic methods, previous complicated obstetrical admission, surgical timing (daytime vs. after hours and weekends), and teaching hospital status. Antenatal diagnosis of PAS was determined either by the presence of a physician billing code in the 9 months preceding delivery for predelivery magnetic resonance imaging (MRI) alone or in combination with maternal fetal medicine (MFM) specialist consultation (see Supporting Information: Appendix B).

### 2.3 Statistical analysis

To evaluate trends in the provincial volume of PAS hysterectomies and the number of distinct institutions performing PAS hysterectomies, we fitted saturated Poisson regression models of the annualized count of each as a function of the calendar year. We took a *P* value of less than 0.05 from the likelihood ratio test from these models as indicative of a statistically significant temporal trend of the modeled count. Additionally, we evaluated temporal trends in average institutional PAS hysterectomy volume and the probability of the primary outcome using linear regression and the Cochran‐Armitage test, respectively.

We compared outcomes between high‐ and low‐volume centers using modified Poisson regression with a robust sandwich‐type variance estimator and a random intercept to account for outcome clustering at the level of the institution. We adjusted for relevant patient and institutional factors. We treated institutional volume as both dichotomized (where high‐volume was ≥9 PAS cases) and as a continuous variable to assess its relationship with the probability of severe maternal outcomes. We also performed a sensitivity analysis where we repeated the main analysis using a lower cutoff of ≥6 PAS cases in the prior year to define high‐volume centers.

## 3 RESULTS

We included 748 patients who underwent cesarean hysterectomy for PAS during the accrual window (Figure S3).

### 3.1 Trends in PAS care over time

Temporal analysis of PAS hysterectomy cases showed an increase in the number of procedures performed over the study period, with 27 cases performed in 2003 compared to 63 cases in 2021 (Figure S4). There was a statistically significant increase in the incidence of PAS hysterectomy over time (*P* < 0.001).

The number of institutions that performed PAS hysterectomy was stable (range: 13–24 institutions per year) over the study period (Figure S5). PAS hysterectomy was performed by 74 unique institutions (Table S1), three of which were designated as high‐volume (i.e., performing ≥9 PAS hysterectomies in the prior year).

**FIGURE 1:**
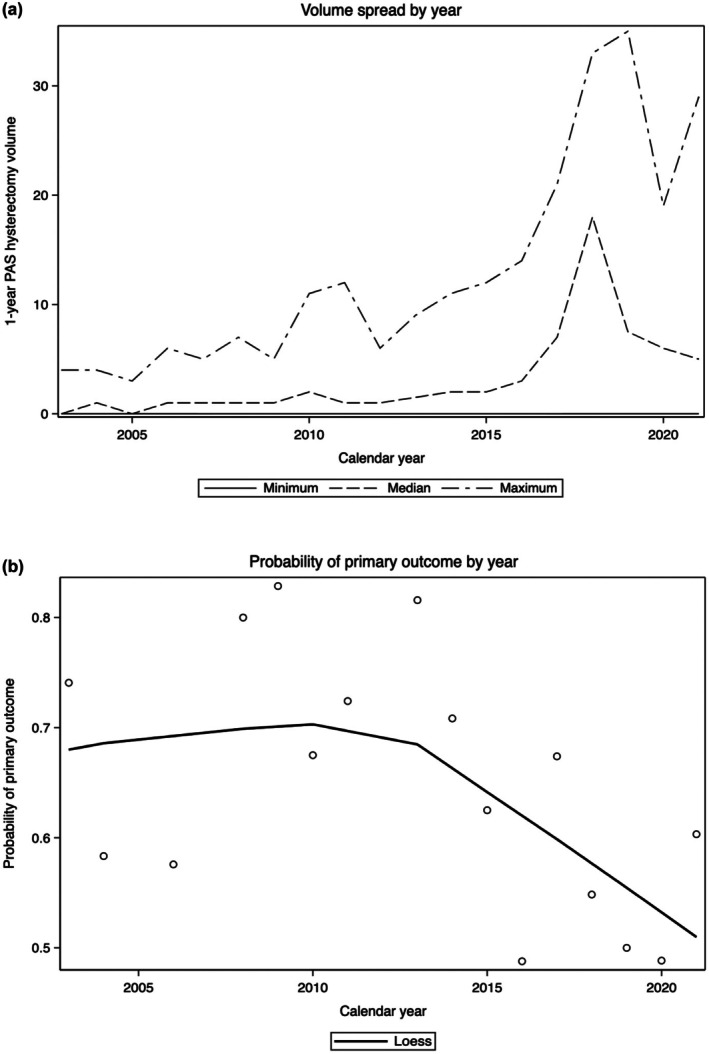
(a) Institutional volume spread for placenta accreta spectrum (PAS) hysterectomies over time. (b) Probability of severe maternal morbidity over time.

The spread in the volume of hysterectomies performed at each center increased over time. In 2003 the highest volume center performed ≤5 hysterectomies in the prior year whereas in 2019 the highest volume center performed 34 cases in the prior year and the lowest volume center performed 0 cases (Figure 1a). Prior to 2010, all institutions in Ontario were classified as low volume.

Trends in the probability of SMM were evaluated over time and demonstrated a statistically significant decrease in the probability of maternal morbidity over the study period (*P* < 0.001; Figure 1b).

### 3.2 Baseline characteristics in high versus low volume institutions

Of the 748 cases performed, 151 were performed at high‐volume institutions and 597 cases were performed at low‐volume institutions. Table 1 outlines the baseline characteristics of patients undergoing surgery at high‐ and low‐volume institutions and shows slight differences in patient profiles.

**TABLE 1:** (a) Baseline sociodemographic and prior health characteristics by volume. (b) Baseline current pregnancy characteristics.

Variable	Total (*N* = 748)	High (*N* = 151)	Low (*N* = 597)	Standardized difference high vs. low	*P* value high vs. low
(a) Age at index date					
Mean (SD)	34.28 (5.00)	35.05 (4.41)	34.08 (5.12)	0.20	0.03
Census based neighborhood income quintile					
First	180 (24.1%)	34 (22.5%)	146 (24.5%)	0.05	0.24
Second	141 (18.9%)	24 (15.9%)	117 (19.6%)	0.10	
Third	145 (19.4%)	36 (23.8%)	109 (18.3%)	0.14	
Fourth	147 (19.7%)	24 (15.9%)	123 (20.6%)	0.12	
Fifth	130–134^a^	28–32^a^	97–101^a^	0.11	
Missing	1–5^a^	1–5^a^	1–5^a^	0.08	
Rurality					
Missing	11 (1.5%)	1–5^a^	6–10^a^	0.05	0.10
Rural	47 (6.3%)	2–6^a^	41–45^a^	0.21	
Urban	690 (92.2%)	144 (95.4%)	546 (91.5%)	0.16	
Weighted baseline ADG summary score					
Median (SD)	−11.15 (8.80)	−9.21 (8.94)	−11.64 (8.70)	0.28	<0.01
Sum of major ADGs					
0	372 (49.7%)	56 (37.1%)	316 (52.9%)	0.32	<0.01
1	241 (32.2%)	60 (39.7%)	181 (30.3%)	0.20	
2	86 (11.5%)	23 (15.2%)	63 (10.6%)	0.14	
3+	49 (6.6%)	12 (7.9%)	37 (6.2%)	0.07	
Parity					
0	202 (27.0%)	26 (17.2%)	176 (29.5%)	0.29	<0.01
1	267 (35.7%)	55 (36.4%)	212 (35.5%)	0.02	
2	188 (25.1%)	40 (26.5%)	148 (24.8%)	0.04	
3+	91 (12.2%)	30 (19.9%)	61 (10.2%)	0.27	
Previous induced abortion					
0	576 (77.0%)	117 (77.5%)	459 (76.9%)	0.01	0.14
1	129 (17.2%)	21 (13.9%)	108 (18.1%)	0.11	
2+	43 (5.7%)	13 (8.6%)	30 (5.0%)	0.14	
Obesity	65 (8.7%)	17 (11.3%)	48 (8.0%)	0.11	0.21
Pre‐existing diabetes	26 (3.5%)	1–5^a^	21–25^a^	0.06	0.53
(b) Mode of delivery					
Missing	211 (28.2%)	10–14^a^	197 (33.0%)	0.61	<0.001
Cesarean	518 (69.3%)	136 (90.1%)	382 (64.0%)	0.65	
Assisted vaginal	14–18^a^	0 (0.0%)	13–17^a^	0.22	
Vaginal	1–5^a^	1–5^a^	1–5^a^	<0.01	
Gestational age at delivery					
Missing	73 (9.8%)	4–8^a^	65–69^a^	0.31	<0.001
<24–27^+6^	29 (3.9%)	2–6^a^	23–27^a^	0.04	
28–31^+6^	51 (6.8%)	13 (8.6%)	38 (6.4%)	0.09	
32–35^+6^	238 (31.8%)	80 (53.0%)	158 (26.5%)	0.56	
36+	357 (47.7%)	48 (31.8%)	309 (51.8%)	0.41	
Antenatal diagnosis	331 (44.3%)	137 (90.7%)	194 (32.5%)	1.50	<0.001
Antenatal diagnosis with consult	151 (20.2%)	98 (64.9%)	53 (8.9%)	1.43	<0.001
Previous complicated^a^ OB admission	203 (27.1%)	58 (38.4%)	145 (24.3%)	0.31	<0.001
Hypertensive disorder of pregnancy	53 (7.1%)	6 (4.0%)	47 (7.9%)	0.17	0.10
After‐hours emergency case	355 (47.5%)	50 (33.1%)	305 (51.1%)	0.37	<0.001
Teaching hospital	482 (64.4%)	151 (100.0%)	331 (55.4%)	1.27	<0.001

Abbreviations: ADG, aggregated diagnosis group; SD, standard deviation.

^a^ Complicated admission includes admission for antepartum hemorrhage, morbidly adherent placenta, placental abruption and false labor.

Notably, patients treated at high‐volume institutions were more likely to have had an antenatal diagnosis of PAS and have received a MFM antenatal consult prior to surgery. High‐volume centers were more likely to perform elective procedures and had fewer after‐hours emergency cases. All high‐volume centers were university‐affiliated medical centers engaged in teaching.

### 3.3 Primary outcome

Sixty‐eight patients (45.0%) at high‐volume centers experienced severe maternal morbidity, compared to 428 (71.7%) at low volume centers (Table S2). This translated to a 41% risk reduction after adjusting for baseline covariates (adjusted relative risk [aRR] 0.59, 95% confidence interval [CI]: 0.49–0.72).

### 3.4 Secondary outcomes

Patients operated on at high‐volume centers had a 43% decreased risk of massive transfusion (43.0% vs. 70.2%; aRR 0.57, 95% CI: 0.47–0.70), a 40% decreased risk of any transfusion (45.0% vs. 70.2%; aRR 0.60, 95% CI: 0.49–0.73), and a 70% decreased risk of maternal ICU admission (7.3% vs. 31.3%; aRR 0.30, 95% CI: 0.15–0.58). Patients operated on at high‐volume centers also had a significantly shorter length of hospital stay after surgery (1.88 days vs. 3.90 days; *P* < 0.001). After adjusting for within‐institution correlation, patients treated in high‐volume centers were 61% more likely to undergo UAE in the perioperative period (aRR 0.39, 95% CI: 0.35–0.45). There were no significant differences for embolism/shock/DIC or return to hospital. Risks for several other secondary outcomes, including death and surgical complications, could not be calculated due to zero or small event counts.

Analyzing institutional volume as a continuous variable (Table S3) demonstrated that each additional PAS hysterectomy performed in the previous year was associated with an average reduction in the risk of the primary composite outcome of 3% (aRR 0.97, 95% CI: 0.96–0.98). We also found a statistically significant reduction in the risk of massive transfusion (aRR 0.96, 95% CI: 0.95–0.97) and any transfusion (aRR 0.96, 95% CI: 0.95–0.97). Each additional hysterectomy performed by a center in the prior year corresponded to a 14% reduction in the risk of return to the operating room (aRR 0.86, 95% CI: 0.78–0.95) and a 9% reduction in the risk of maternal ICU admission (aRR 0.91, 95% CI: 0.87–0.95). Patients also experienced a statistically significant reduction in length of stay for each additional hysterectomy case performed at that institution in the previous year (aRR 0.97, 95% CI: 0.97–0.97).

### 3.5 Sensitivity analyses

Sensitivity analysis using a lower threshold of ≥6 PAS hysterectomies to define high‐volume centers yielded similar but attenuated results for both primary and secondary outcomes (Table S4).

## 4 DISCUSSION

In this population‐based cohort study examining hysterectomies performed for PAS in the province of Ontario, we found a rising incidence of PAS cases over the study period alongside a significant reduction in PAS related SMM over the same period. Unlike prior single‐center or registry‐based studies, this design captured all PAS hysterectomies performed across Ontario within a universal health system, enabling adjustment for both patient‐ and institution‐level covariates and enhancing generalizability.

While the number of institutions performing PAS hysterectomies by year remained stable (13 to 24 centers per year), three institutions emerged as high‐volume centers, defined as performing at least nine PAS hysterectomies annually. Over time, a greater proportion of patients were treated at these high‐volume centers, which demonstrated improved outcomes, including a 41% reduction in SMM. Patients at high‐volume centers were less likely to require massive transfusions, ICU admissions, or prolonged hospital stays. Furthermore, as institutional PAS volume increased, the need to return to the operating room decreased, while UAE increased.

These findings are consistent with and extend those from prior reports,^12^ emphasizing the critical role of multidisciplinary teams and specialized care in improving outcomes for patients with PAS. Several studies have shown that patients treated by dedicated PAS teams in high‐volume centers experience significantly better outcomes, including reduced blood loss, fewer transfusions, and lower risks of ureteric injury and reoperation.^4^, ^5^, ^6^, ^7^, ^12^ Multiple consensus statements and guidelines underscore the importance of multidisciplinary care in centers of excellence for optimizing PAS outcomes.^5^, ^17^, ^18^, ^19^, ^20^, ^21^, ^22^, ^23^, ^24^

Despite these consensus recommendations, our study revealed that 74 unique institutions performed PAS hysterectomies, where only three were high‐volume centers. This suggests a need for better prenatal screening and diagnosis that can facilitate the centralization of PAS care to multidisciplinary teams with protocolized management to deliver improved patient outcomes.^9^, ^25^ The rise in PAS cases over the past decade underscores the urgent need to work towards decreasing risk factors, including elective cesarean delivery,^26^ and optimizing referral pathways. While the increased incidence may partly reflect improved detection through better diagnostic methods, the risk profile of the pregnant population, especially the proportion becoming pregnant following prior uterine surgery, has increased.

When considering regionalized care for PAS, it is essential to include patient perspectives. Regionalization challenges patients and their families to either relocate to urban centers before and after surgery, or to leave other children at home under alternate care arrangements for extended periods prior to birth. These logistical challenges must be balanced with the evidence that regionalized care is associated with a significant reduction in SMM for patients in high‐volume centers, but this evidence should not be ignored. Prioritizing a patient‐centered approach is key, but it is also crucial to weigh the high‐risk nature of this relatively uncommon surgery. From a systems perspective, regionalizing PAS care offers considerable benefits, similar to the regional cancer care model, enhancing patient safety and outcomes.

The improved outcomes observed in high‐volume centers may be attributed to several factors, including the presence of dedicated maternal‐fetal medicine specialists and pelvic surgeons who coordinate a high level of diagnostic accuracy and interdisciplinary care, integrating themselves with the necessary surgical expertise in complex pelvic surgery, including the skills for internal iliac artery dissection and ligation, and the availability of intraoperative cell‐salvage, comprehensive transfusion medicine and ICU support.^27^, ^28^, ^29^, ^30^ High‐volume centers were also more likely to perform elective, rather than emergent, surgeries, which tend to be associated with better preoperative planning and outcomes since more surgeries will be performed by a specialized multidisciplinary team.^8^ The higher rate of antenatal diagnosis and MFM consultation in the high‐volume group (90.7% vs. 32.5%) underscores the likelihood that early detection and referral contribute to improved outcomes. It is likely that some of the cases at very low‐volume centers present undiagnosed or actively bleeding, requiring emergent intervention, naturally predisposing them to suboptimal outcomes. The discrepancy in outcomes between high‐ and low‐volume institutions points to the critical need principally to improve early prenatal screening and diagnosis, such that local providers, patients and their families can make informed decisions regarding centralized versus local care or alternatively that lower‐volume centers collaborate with their regional center to adopt standardized protocols developed by high‐volume, specialized centers.^25^, ^31^ Centralization may reduce the burden on low‐volume centers and allow for more consistent, high‐quality care across institutions.

Future research should aim to identify the specific elements within high‐volume centers that drive improved maternal outcomes for PAS hysterectomy. Future research should also explore health equity implications, ensuring that improvements in PAS care are accessible to all patient populations, including those in low‐resource settings.

The present study's strength lies in its comprehensive, population‐based design over a 19‐year period, which captures Ontario's diverse demographics within a single‐payer fee‐for‐service publicly funded healthcare system. This setting minimizes economic disparities in patient care that are known to affect surgical outcomes. Additionally, the longitudinal nature of the data enables both cross‐cohort comparisons (high‐ vs. low‐volume) and trend analysis over time.

These data must be interpreted in the context of the study design. Retrospective data may be prone to selection bias and residual confounding may persist despite adjustments. Outcomes could be influenced by institutional variations in protocols that were not accounted for in our analysis. Furthermore, administrative databases may contain errors and lack granular details such as the extent of placental invasion, individual surgical case complexity, the presence of multidisciplinary teams, and patient preferences. Although antenatal diagnosis and timing of surgery (including after‐hours cases) were adjusted for in our analyses, unmeasured factors related to these variables may still contribute to residual confounding. Because our use of administrative data did not allow for classification of placental invasion severity according to the FIGO grading system, we were unable to differentiate outcomes by PAS subtype (e.g., accreta, increta, percreta) or depth of invasion. However, given that all included cases underwent hysterectomy, it is reasonable to assume that this cohort represents the most severe phenotypes of PAS requiring definitive surgical management.

## 5 CONCLUSION

Our study underscores the critical role of high‐volume, specialized centers in enhancing maternal outcomes for patients with PAS disorders. By demonstrating significantly lower rates of severe maternal morbidity, reduced need for massive transfusion, shorter hospital stays, and fewer ICU admissions in high‐volume institutions, our findings strongly advocate for a centralized approach to PAS care.

A multidisciplinary model that includes experienced obstetric surgeons, maternal‐fetal medicine specialists, anesthesiologists, interventional radiologists, and critical care teams is likely key to these improved outcomes. High‐volume centers may benefit from enhanced surgical proficiency, standardized management protocols, advanced blood conservation techniques, and improved perioperative coordination, all of which contribute to optimizing patient safety.

Given the complexity and life‐threatening nature of PAS, our findings support the need for regionalized care networks that can facilitate timely referrals to high‐volume institutions. Establishing PAS centers of excellence could ensure that patients receive evidence‐based, coordinated care while potentially reducing healthcare costs associated with complications and prolonged hospital stays.

## AUTHOR CONTRIBUTIONS

L.C.B.: Contributed to conceptualization, study design, data interpretation, manuscript drafting, and critical review. O.B., P.S.T., L.A., and S.R.H.: Contributed to conceptualization, data interpretation, and manuscript review. J.S.: Contributed to study design, statistical analysis, data management, and critical review. J.P.: Contributed to conceptualization, study design, statistical oversight, and manuscript review. J.K.: Provided clinical expertise and contributed to conceptualization, data analysis, and manuscript review. M.P.V. Contributed to conceptualization, supervision, data interpretation, and manuscript review. A.M. Contributed to conceptualization, study design, data interpretation, study oversight, and critical revisions.

## FUNDING INFORMATION

We would like to thank the Department of Obstetrics and Gynecology at Sinai Health, Toronto, Ontario, the University of Toronto, and the Joseph M. West Family Memorial Fund for their support of this study. In addition, this study was supported by ICES, which is funded by an annual grant from the Ontario Ministry of Health (MOH) and the Ministry of Long‐Term Care (MLTC). Parts of this material are based on data and/or information compiled and provided by CIHI and the Ontario MOH. The analyses, conclusions, opinions and statements expressed herein are solely those of the authors and do not reflect those of the funding or data sources; no endorsement is intended or should be inferred. This document used data adapted from the Statistics Canada Postal Code Conversion File, which is based on data licensed from Canada Post Corporation, and/or data adapted from the Ontario Ministry of Health Postal Code Conversion File, which contains data copied under license from Canada Post Corporation and Statistics Canada.

## CONFLICT OF INTEREST STATEMENT

O.B. reports serving on the advisory board and speaker bureau for Abbvie and Pfizer as well as the data safety monitoring board for Organon. The remaining authors have nothing to disclose.

## Supporting information


Appendix A‐C.

Data S1.

## Data Availability

Research data are not shared.
