# Supplementary material for: Hysterectomy for placenta accreta spectrum disorder: Impact of institutional surgical volume on patient outcomes
Source: Int J Gynaecol Obstet. 2026 Feb 4;174(1):382–90. doi: 10.1002/ijgo.70846 (PMC13278653; doi:10.1002/ijgo.70846)
Supplement: Supplementary file 1 — Appendix A‐C. [file IJGO-174-382-s001.docx]

**Appendix A.** Dataset descriptions.

| **Database Name** | **Acronym** | **Description** | **References (if applicable)** |
| --- | --- | --- | --- |
| **Health Services** | | |  |
| Discharge Abstract Database | DAD | This dataset contains patient-level data for acute, rehab, chronic, and day surgery institutions in Ontario. Captures administrative, clinical, and demographic information. About 85% of all hospital inpatient discharges in Canada are captured in DAD. Notable exclusions from DAD include mental health admissions and patients awaiting a bed in a long-term care facility who are designated as ‘alternate level of care’. |  |
| National Ambulatory Care Reporting System | NACRS | Contains data for all hospital-based and community-based ambulatory care. Including outpatient and community-based clinics and emergency departments. Captures administrative, clinical, and demographic information. A small number of ambulatory clinics on federally funded reserves in Ontario do not submit ambulatory care data. |  |
| Ontario Health Insurance Plan Billing Database | OHIP | Contains data on physician billing claims paid by the Ontario Health Insurance Plan. The data cover all health care providers who can claim under OHIP (this includes physicians, groups, laboratories, and out-of-province providers). OHIP data accurately reflects the utilization of physician services in Ontario (only a few hundred family physicians who are remunerated through a mechanism other than OHIP). Captures information on the code for service provided, dated of service, associated diagnosis and fee paid. |  |
| Same Day Surgery | SDS | This dataset contains patient-level data for day surgery institutions in Ontario. Every record corresponds to one same-day surgery or procedure stay.  Captures administrative, clinical, and demographic information. Same Day Surgery is defined as, All procedures performed on patients whose hospitals stay/visit (from time of registration to discharge), occurs on the same calendar day or, if over the midnight hour, is less than 12 hours, irrespective of the site/location within the hospital, anaesthetic route/administration, and whether the procedure was scheduled/unscheduled. |  |
| **Population and Demographics** | | |  |
| Registered Persons Database | RPDB | A dataset that provides demographic information about all individuals who have received an Ontario health card number, including their date of birth, sex, and home address. |  |
| Postal Code Conversion File | PCCF | A digital file which provides a correspondence between the Canada Post Corporation (CPC) six-character postal code and Statistics Canada’s standard geographic areas for which census data and other statistics are produced. |  |
| **Facilities** | | |  |
| Institution Information System | INST | A series of linkable datasets containing information about Ontario health care institutions funded by the Ministry of Health and Long-Term Care (MOHLTC). |  |
| **ICES Derived Cohorts** | | |  |
| Ontario Hypertension Dataset | HYPER | A dataset of all of the people in Ontario identified as having hypertension using a validated algorithm. | <https://pubmed.ncbi.nlm.nih.gov/20101286/>  <https://doi.org/10.1503/cmaj.071283> |
| Mother-Baby Linked Dataset | MOMBABY | This dataset links the DAD inpatient admission records of delivering mothers and their newborns. Each record corresponds to a mother-child pair. The sensitivity and specificity of the algorithm linking mother and newborn records are 0.9613 and 0.9924 for years from 2002/03 to 2009/10 respectively. |  |
| Ontario Diabetes Dataset | ODD | A dataset of all of the people in Ontario diagnosed with diabetes using a validated algorithm. | <https://doi.org/10.1186/s12913-018-3148-0>  <https://doi.org/10.1111/j.1399-5448.2009.00539.x>  <https://doi.org/10.2337/diacare.25.3.512> |

**Appendix B.** Cohort creation description.

| **Concept** | **Procedure/Condition/Concept** | **Data Sources** | **Code Type** | **Window** | **Applicable Codes** |
| --- | --- | --- | --- | --- | --- |
| Inclusion Criteria | Hysterectomy of any type (total, radical, subtotal) by any route (laparoscopic assisted vaginal, vaginal, laparoscopic, open), or cesarean hysterectomy | CIHI-DAD, SDS | CCI | Ref date = index date | 1RM89AA, 1RM89CA, 1RM89DA, 1RM89LA, 1RM91AA, 1RM91CA, 1RM91DA, 1RM91LA, 1RM87LA, 1RM87DA, 5.MD.60.RC, 5.MD.60.RD, 5.MD.60.KE, 5.MD.60.CB |
|  | Diagnosis of morbidly adherent placenta (accreta, increta or percreta.) | CIHI-DAD, SDS | ICD-10-CA | Ref date = index date | O43.21, O43.22, O43.23 |
| Exclusion Criteria | Missing or invalid IKN | RPDB |  |  | Variable VALIKN not equal to “V” |
|  | Missing or invalid age (<15 or >50) | RPDB |  | Ref date = index date | Age at index derived from variable BDATE |
|  | Death before the index date | RPDB |  | - | Variable DTHDATE is earlier than index date |
|  | Non-Ontario residents | RPDB |  | Ref date = index date | First two digits of the province-census dissemination identifier variable (PR\|CD\|DA\|BLK) not equal to “35” |
|  | Male or missing sex | RPDB |  |  | Variable SEX not equal to “F” |
|  | Unable to identify institution | INST |  |  | Missing INSTNUM |
|  | Previous Hysterectomy | CIHI-DAD, SDS | CCI, CCP | 10 year look back | CCI: 1.RM.89.AA, 1.RM.89.CA, 1.RM.89.DA, 1.RM.89.LA, 1.RM.91.AA, 1.RM.91.CA, 1.RM.91.DA, 1.RM.91.LA, 1.RM.87.LA, 1.RM.87.DA, 5.MD.60.RC, 5.MD.60.RD, 5.MD.60.KE, 5.MD.60.CB  CCP: 802, 803, 804, 805, 806 |
| Baseline Sociodemographic and Prior Health Characteristics | Age at index date | RPDB |  | Ref date = index date |  |
|  | Neighbourhood Income Quintile | RPDB |  | Ref date = index date | Field INCQUINT |
|  | Rurality | RPDB |  | Ref date = index date | Variable RIO2008 |
|  | Weighted Baseline ADG Summary Score | DAD, SDS, NACRS, OHIP | All records and claims | Within 2 years prior to index date | Computed using version 10 of the Johns Hopkins ACG® System (Reference: <https://doi.org/10.1097/mlr.0b013e318229360e> ) |
|  | Sum of Major ADGs | DAD, SDS, NACRS, OHIP | All records and claims | Within 2 years prior to index date |  |
|  | Parity | MOMBABY |  | Ref date = index date | Presence of any record with a delivery date prior to index |
|  | Previous Induced Abortions | DAD, SDS | ICD-10-CA,  CCI, CCP | Ref date = index date | ICD-10-CA: O04, O08  CCI: 5.CA.88.*, 5.CA.89.*, 5.CA.20FK, 5.CA.24.*  CCP: 810.1, 870, 871, 872.1, 872.9 |
|  | Obesity | OHIP | Fee code, OHIP dxcode | Within 2 years prior to index date | Fee codes: E676A, E676B, E010C  OHIP dxcode: 278 |
|  | Pre-existing Diabetes | ODD |  | Ref date = index date | Variable DXDATE prior to index date |
| Baseline Current Pregnancy Characteristics | Mode of Delivery (spontaneous vaginal, assisted vaginal, cesarean delivery, cesarean hysterectomy) | CIHI-all | CCI, CCP | Within 30 days prior or equal to index date. | 5.MD.51, 5.MD.56, 5.MD.52, 5.MD.53, 5.MD.54, 5.MD.55, 5.MD.60. 5.MD.60.RC, 5.MD.60.RC, 5.MD.60.RD, 5.MD.60.KE, 5.MD.60.CB |
|  | Gestational age at delivery | MOMBABY |  |  | Field M_GESTWKS_DEL |
|  | Antenatal Diagnosis by MRI | OHIP | Fee code | Within 9 months prior to index date | X451, X455, X461, X465 |
|  | Antenatal Diagnosis + MFM Consult | OHIP | Fee code | Within 9 months prior to index date | Any of: (X451, X455, X461, X465)  AND P002 |
|  | Previous Complex Obstetrical Admission (hemorrhage, morbidly adherent placenta, previa, abruption, false labour) | DAD | ICD-10 | Discharge date within 9 months prior to 1 day prior to index | O72.0, O43.2x, O44, O45, O46, O47 |
|  | Hypertensive Disorders in Pregnancy | CIHI-all | ICD-10 | Within 9 months prior to index date | O10, O11, O13, O14, O15, O16 |
|  | After-hours emergency case (Evenings, Nights, Weekends) | OHIP | Fee code | Ref date = index date | E409, E410, E400, E401 |
|  | Teaching hospital status | INST |  |  | Field HOSPTYPE |

**Appendix C.** Outcome description.

| **Outcome** | **Data Sources** | **Code Type** | **Window** | **Applicable Codes** |
| --- | --- | --- | --- | --- |
| Severe Maternal Morbidity (SMM) Composite Outcome | RPDB  DAD  SDS  NACRS  OHIP |  | Within 42 days after index | Include codes for: death, ICU admission, embolism/shock/DIC, surgical complications, sepsis, |
| Massive transfusion | DAD |  | Within 42 days after index | BTANY = 1 or Y + any of: BTPLATE, BTPLASMA, BTALBUM, BTPPC, BTFIBRINOGEN, BTCSPLASMA, BTCPPLASMA, BTANTIT3, BTCPROTHER, BTOTHER |
| Any transfusion | DAD |  | Within 42 days after index | BTANY = 1 or Y |
| Intensive care unit admission | DAD | ICD-10-CA | Within index date + 1 day to index + 42 days after index | SCU1-SCU6 with any of the listed types of intensive care units (10, 20, 25, 30, 40, 45, 60, 80) =Y, on any other day than the index date and within 30 days of surgery. |
| Uterine artery embolization | CCI |  | Within index date + 1 day to index + 42 days after index | 1.RM.13 |
| Return to ED/ hospital | NACRS  DAD |  | Within 42 days after index | Any unscheduled ED visits in NACRS  Field ENTRY = C, D, or E in DAD |
| Shock/DIC/Embolism | DAD | ICD-10-CA | Within 42 days after index | R57, O751, D65, D688, D689, O88, I26, I82, I80, O22.3, O22.9 |
| Surgical complications (Urological, intestinal, vascular) | DAD | ICD-10-CA  CCI | Within 42 days after index | ICD-10-CA: S371, S372, S373, S364, S365, S366, S367, S368, S35  Cci: 1.NK.80.*, 5.PC.80.JR, 1.NM.80.* |
|  |  |  |  |  |
|  |  |  |  |  |
| Unplanned Return to OR | DAD |  | Within 42 days after index | Nonmissing value in INUNPL1-INUNPL20 NE during index admission. |
| Sepsis | DAD | ICD-10-CA | Within 42 days after index | A41, A40, A021, A227, A267, A327, A427, A5486, B377, R65, O85, O75.3 |
| Acute renal failure | DAD | ICD-10-CA  CCI | Within 42 days after index | ICD-10-CA: N17, N19, N990, O904  CCI: 1.PZ.21 |
| Cardiac complications | DAD | ICD-10-CA  CCI | Within 42 days after index | ICD-10-CA: R09.2, I46, I47, I48, I21, I22, I23, I50, J81, O.90.3, I42, I43, O89.1  CCI: 1.HZ.09, 1.HZ.30 |
| Death | RPDB  DAD |  | Within 42 days after index | Dthdate on RPDB  Death during index hospitalization: DischargeDisposition=7 (DAD)  Death in hospital (after index hospitalization): DischargeDisposition=7 at subsequent hospitalization within 42 days (DAD)  Death outside hospital (after index hospitalization):  Dthdate, not covered above |
| Cerebrovascular complications | DAD | ICD-10-CA | Within 42 days after index | H34, I63, I64, I61, I62, I60, G45, R402, O225, O873 |
| Length of stay following index | DAD  SDS |  | Ref date = index date | ‘DDATE’ minus ‘Index Date’ in days  (from OR date to discharge including transfers); length of stay recorded as 1 if discharged on the same day as the OR date. |
