# Supplementary material for: Hysterectomy for placenta accreta spectrum disorder: Impact of institutional surgical volume on patient outcomes
Source: Int J Gynaecol Obstet. 2026 Feb 4;174(1):382–90. doi: 10.1002/ijgo.70846 (PMC13278653; doi:10.1002/ijgo.70846)
Supplement: Supplementary file 2 — Data S1. [file IJGO-174-382-s002.docx]

**SUPPLEMENTAL DATA**

1. **eFigure 1.** Predicted probability of SMM (primary outcome) by fractional polynomial-transformed continuous volume.
2. **eFigure 2.** Receiver operating curve analysis for prediction of best dichotomized cutoff for high- and low-volume institutions.
3. **eFigure 3.** Inclusion/exclusion flow chart.
4. **eFigure 4.** Number of PAS hysterectomy cases performed each year in Ontario.
5. **eFigure 5.** Number of institutions performing PAS hysterectomy over time.
6. **eTable 1.** Institutional volume description by decile.
7. **eTable 2.** Outcomes by Volume – main definition (≥9 PAS hysterectomies performed in the prior year).
8. **eTable 3.** Outcomes by volume when volume treated as a continuous variable.
9. **eTable 4.** Outcomes by Volume – sensitivity analysis definition (≥6 PAS hysterectomies performed in the prior year).
10. **Appendix A.** Dataset descriptions.
11. **Appendix B.** Cohort creation description.
12. **Appendix C.** Outcome description.

**eFigure 1.** Predicted probability of SMM (primary outcome) by fractional polynomial-transformed continuous volume.


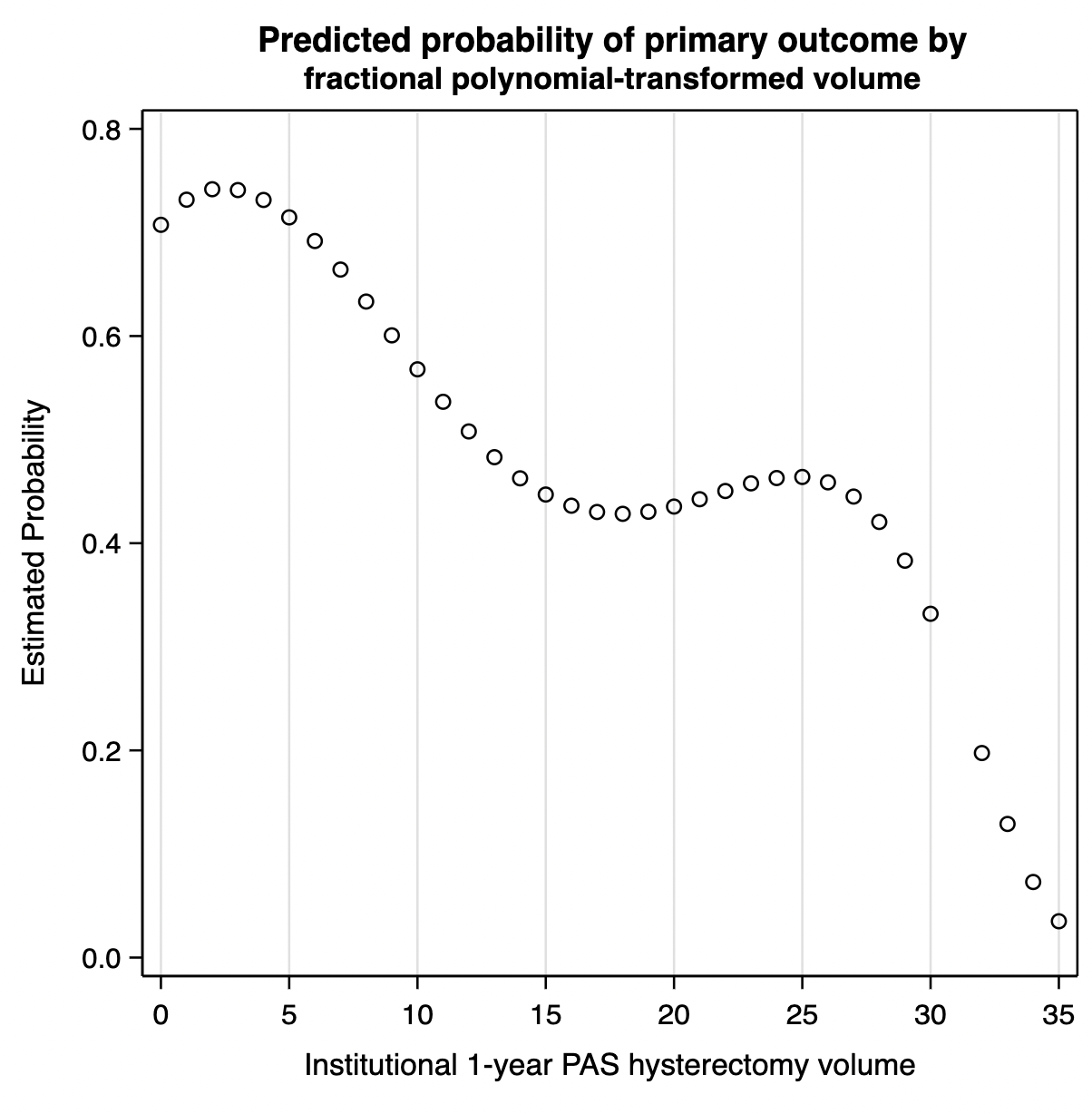


**eFigure 2.** Receiver operating curve analysis for prediction of best dichotomized cutoff for high- and low-volume institutions.


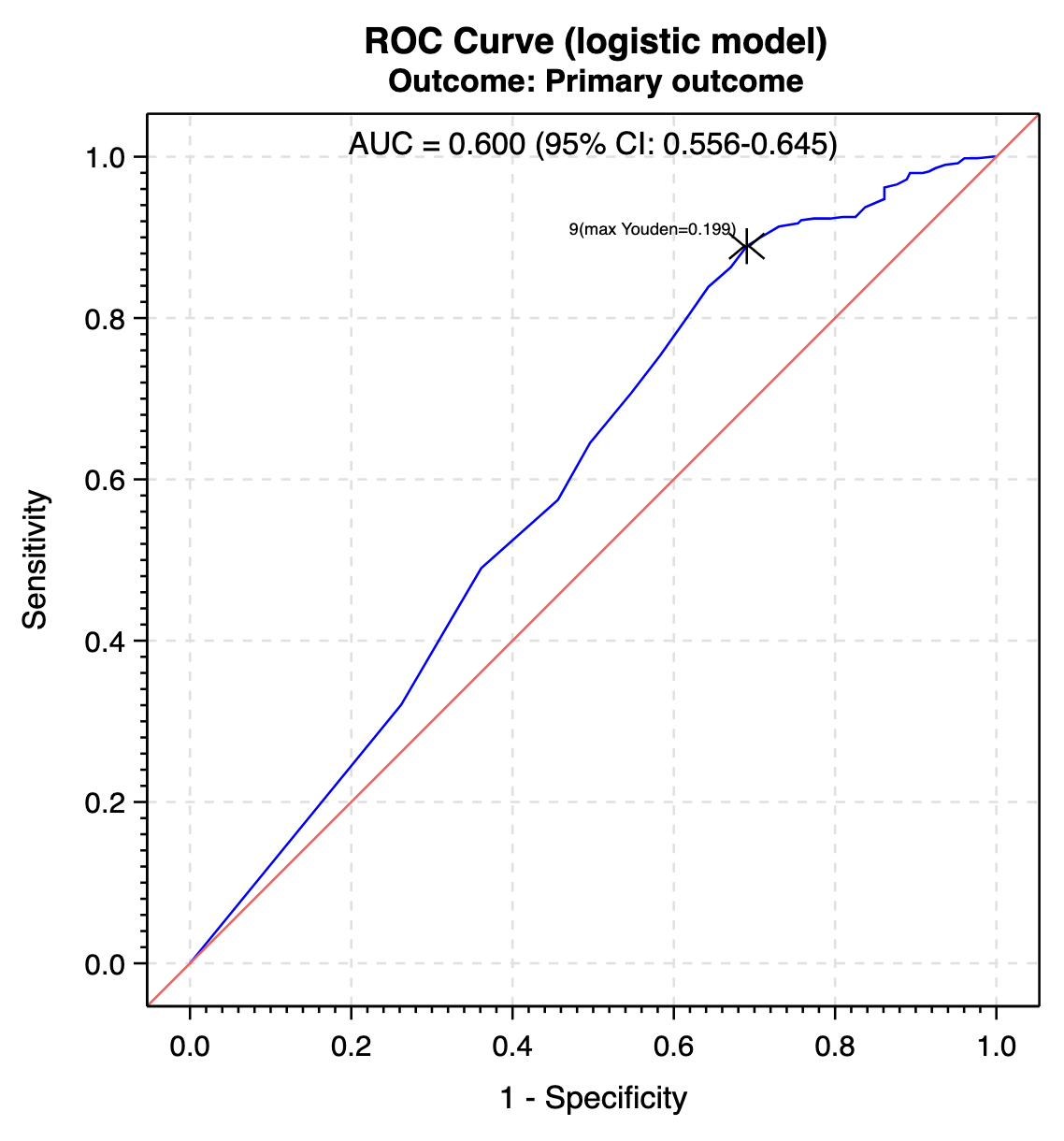


**eFigure 3.** Inclusion/exclusion flow chart.


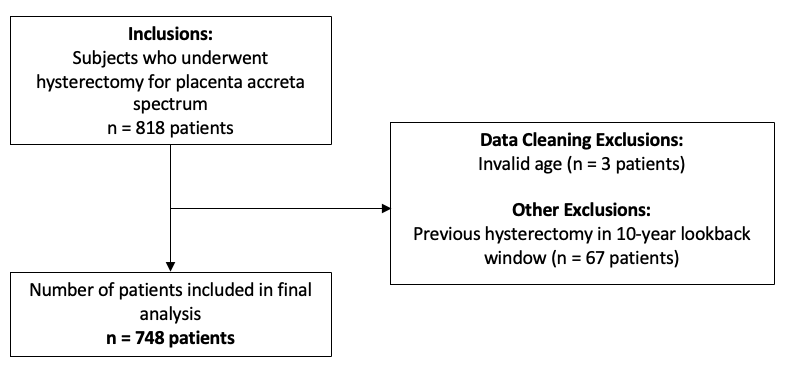


**eFigure 4.** Number of PAS hysterectomy cases performed each year in Ontario. *p for LR test of count = year (categorical; difference between years): < 0.0001. p for simple Poisson regression of count = year (continuous; trend): <0.0001


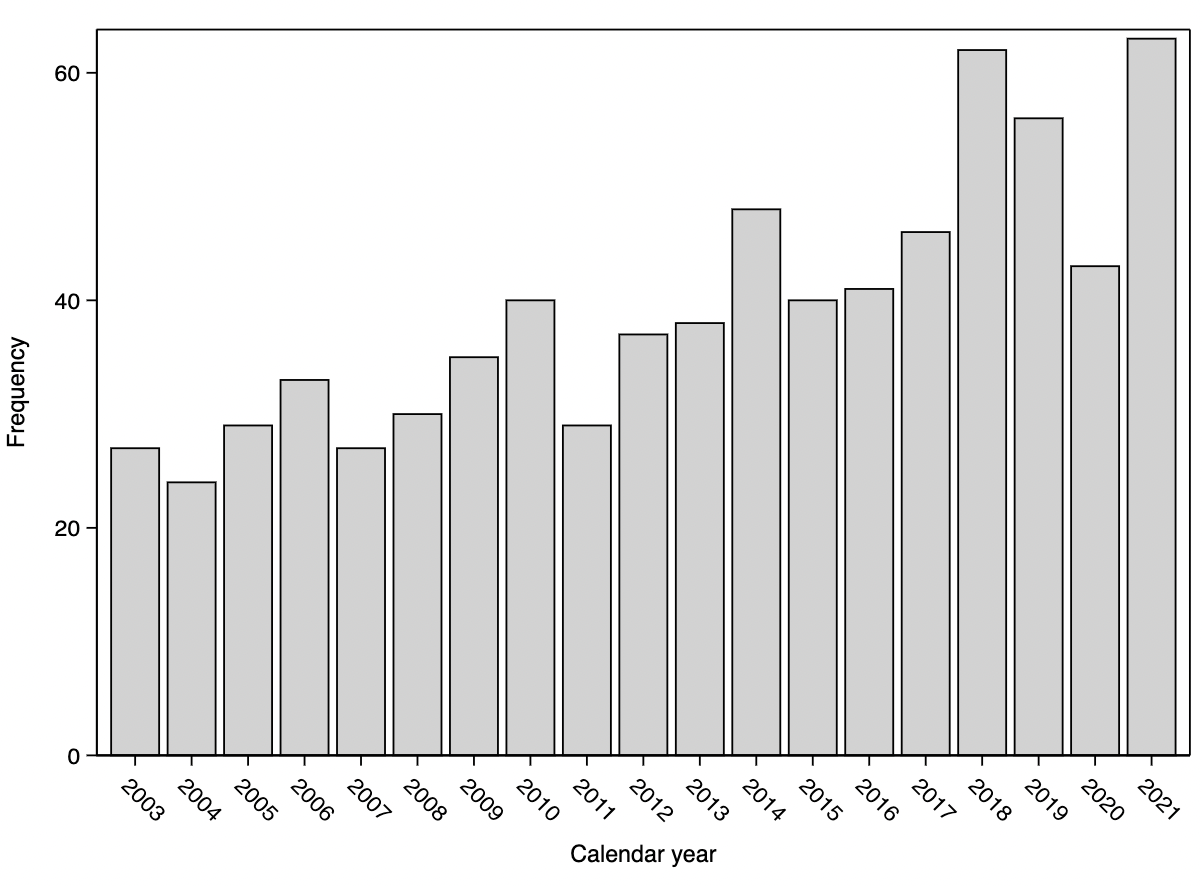


**eFigure 5.** Number of institutions performing PAS hysterectomy over time. *p for LR test of count = year (categorical; difference between years): 0.96; p for simple Poisson regression of count = year (continuous; trend): 0.31.


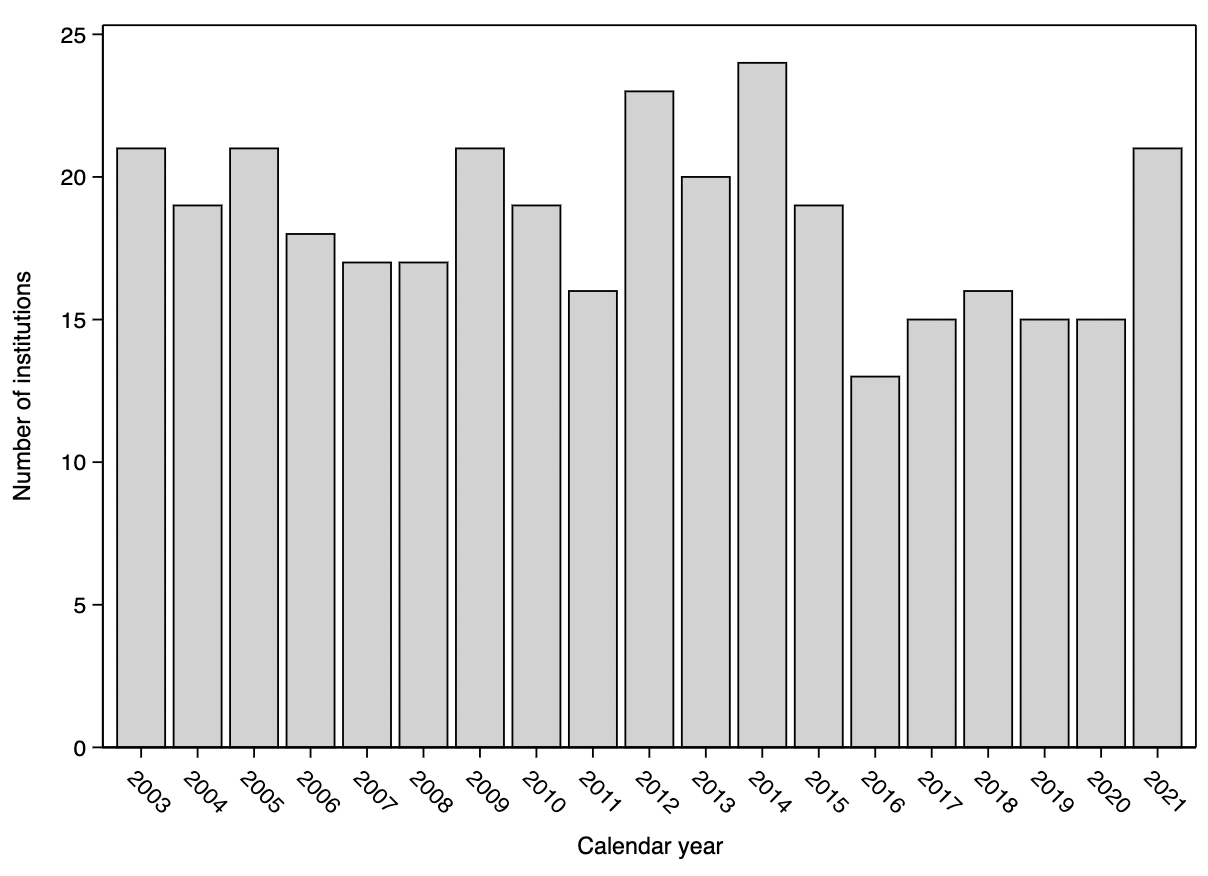


**eTable 1.** Institutional volume description by decile.

| Rank of Institutional Volumes by Decile | N Institutions | # of cases, mean (SD) | # of cases, median (min-max) |
| --- | --- | --- | --- |
| 1 and 2 | 74 | 0.0 (0.0) | 0.0 (0 - 0) |
| 3 and 4 | 36 | 1.0 (0.0) | 1.0 (1 - 1) |
| 5 | 20 | 2.0 (0.0) | 2.0 (2 - 2) |
| 6 | 11 | 3.0 (0.0) | 3.0 (3 - 3) |
| 7 | 9 | 4.4 (0.5) | 4.0 (4 - 5) |
| 8 | 6 | 6.8 (0.8) | 7.0 (6 - 8) |
| 9 | 3 | 12.5 (3.1) | 11.0 (9 - 18) |
| 10 | 1 | 24.7 (4.8) | 24.0 (19 - 35) |

**eTable 2.** Outcomes by Volume – main definition (≥9 PAS hysterectomies performed in the prior year).

| Variable | Total | High | Low | Crude RR (95% CI) | Crude P value | Adjusted RR  (95% CI) | Adjusted P value |
| --- | --- | --- | --- | --- | --- | --- | --- |
| Sample Size | N = 748 | N = 151 | N = 597 |  | . |  | . |
|  |  |  |  |  |  |  |  |
| Primary composite outcome | 496 (66.3%) | 68 (45.0%) | 428 (71.7%) | 0.63 (0.52 - 0.75) | <.0001 | 0.59 (0.49 - 0.72) | <.0001 |
|  |  |  |  |  |  |  |  |
| Massive blood transfusion | 484 (64.7%) | 65 (43.0%) | 419 (70.2%) | 0.61 (0.51 - 0.74) | <.0001 | 0.57 (0.47 - 0.70) | <.0001 |
|  |  |  |  |  |  |  |  |
| Any transfusion | 487 (65.1%) | 68 (45.0%) | 419 (70.2%) | 0.64 (0.53 - 0.77) | <.0001 | 0.60 (0.49 - 0.73) | <.0001 |
|  |  |  |  |  |  |  |  |
| Maternal ICU admission | 198 (26.5%) | 11 (7.3%) | 187 (31.3%) | 0.23 (0.13 - 0.42) | <.0001 | 0.30 (0.15 - 0.58) | 0.0004 |
|  |  |  |  |  |  |  |  |
| Uterine artery embolization | 54 (7.2%) | 17 (11.3%) | 37 (6.2%) | 0.38 (0.36 - 0.41) | <.0001 | 0.39 (0.35 - 0.45) | <.0001 |
|  |  |  |  |  |  |  |  |
| Return to ED/Hospital | 162 (21.7%) | 47 (31.1%) | 115 (19.3%) | 1.62 (1.21 - 2.16) | 0.0012 | 1.21 (0.86 - 1.71) | 0.2808 |
|  |  |  |  |  |  |  |  |
| Embolism/Shock/DIC | 39 (5.2%) | 6 (4.0%) | 33 (5.5%) | 0.80 (0.29 - 2.18) | 0.6600 | 1.56 (0.36 - 6.71) | 0.5502 |
|  |  |  |  |  |  |  |  |
| Surgical complications | 31 (4.1%) | *1-5 | *26-30 | * | . | * | . |
|  |  |  |  |  |  |  |  |
| Return to OR | 22 (2.9%) | *1-5 | *17-21 | * | . | * | . |
|  |  |  |  |  |  |  |  |
| Sepsis | 17 (2.3%) | *1-5 | *12-16 | * | . | * | . |
|  |  |  |  |  |  |  |  |
| Acute renal failure | 7 (0.9%) | *1-5 | *2-6 | * | . | * | . |
|  |  |  |  |  |  |  |  |
| Cardiac complications | 7 (0.9%) | *1-5 | *2-6 | * | . | * | . |
|  |  |  |  |  |  |  |  |
| Death | *1-5 | 0 (0.0%) | *1-5 | N/A | . | N/A | . |
|  |  |  |  |  |  |  |  |
| Cerebrovascular events | 0 (0.0%) | 0 (0.0%) | 0 (0.0%) | N/A | . | N/A | . |
|  |  |  |  |  |  |  |  |
|  | Mean (SD) | | |  |  |  |  |
| Length of hospital stay after index surgery | 4.55 (3.62) | 3.60 (1.88) | 4.79 (3.90) | 0.59 (0.53 - 0.66) | <.0001 | 0.64 (0.58 - 0.71) | <.0001 |

*Rare events including surgical complications, acute renal failure, cerebrovascular events, return to OR and cardiac complications could not be calculated due to zero event counts, and some small-cell results were masked (denoted as “N/A” and “*”, respectively). ICES requires the suppression of all small cells with a count or value of fewer than six (i.e., less than 6) in any publicly released data or publications to protect patient privacy and prevent re-identification.*

**eTable 3.** Outcomes by volume when volume treated as a continuous variable.

| Variable | Total | Crude RR (95% CI) | Crude P value | Adjusted RR (95% CI) | Adjusted P value |
| --- | --- | --- | --- | --- | --- |
| Sample Size | N = 748 |  | . |  | . |
|  |  |  |  |  |  |
| Primary composite outcome | 496 (66.3%) | 0.97 (0.96 - 0.98) | <.0001 | 0.97 (0.96 - 0.98) | <.0001 |
|  |  |  |  |  |  |
| Massive blood transfusion | 484 (64.7%) | 0.97 (0.96 - 0.98) | <.0001 | 0.96 (0.95 - 0.97) | <.0001 |
|  |  |  |  |  |  |
| Any transfusion | 487 (65.1%) | 0.97 (0.96 - 0.98) | <.0001 | 0.96 (0.95 - 0.97) | <.0001 |
|  |  |  |  |  |  |
| Maternal ICU admission | 198 (26.5%) | 0.90 (0.87 - 0.93) | <.0001 | 0.91 (0.87 - 0.95) | <.0001 |
|  |  |  |  |  |  |
| Uterine artery embolization | 54 (7.2%) | 0.88 (0.87 - 0.89) | <.0001 | 0.86 (0.83 - 0.90) | <.0001 |
|  |  |  |  |  |  |
| Return to ED/Hospital | 162 (21.7%) | 1.02 (1.01 - 1.04) | 0.0046 | 1.00 (0.99 - 1.02) | 0.6867 |
| Embolism/Shock/DIC | 39 (5.2%) | 0.97 (0.91 - 1.04) | 0.3797 | 1.02 (0.96 - 1.08) | 0.5848 |
|  |  |  |  |  |  |
| Surgical complications | 31 (4.1%) | 0.97 (0.92 - 1.03) | 0.3320 | 0.94 (0.87 - 1.01) | 0.0980 |
|  |  |  |  |  |  |
| Return to OR | 22 (2.9%) | 0.80 (0.70 - 0.92) | 0.0020 | 0.86 (0.78 - 0.95) | 0.0027 |
|  |  |  |  |  |  |
| Sepsis | 17 (2.3%) | 0.86 (0.81 - 0.92) | <.0001 | 0.88 (0.73 - 1.06) | 0.1732 |
|  |  |  |  |  |  |
| Acute renal failure | 7 (0.9%) | 0.90 (0.74 - 1.11) | 0.3374 | 0.96 (0.88 - 1.04) | 0.3137 |
|  |  |  |  |  |  |
| Cardiac complications | 7 (0.9%) | 0.86 (0.61 - 1.21) | 0.3873 | 1.07 (0.80 - 1.44) | 0.6580 |
|  |  |  |  |  |  |
| Death | *1-5 | * | . | * | . |
|  |  |  |  |  |  |
| Cerebrovascular events | 0 (0.0%) | N/A | . | N/A | . |
|  |  |  |  |  |  |
|  | Mean (SD) |  |  |  |  |
| Length of hospital stay after index surgery | 4.55 (3.62) | 0.97 (0.96 - 0.97) | <.0001 | 0.97 (0.97 - 0.97) | <.0001 |

*Rare events including surgical complications, acute renal failure, cerebrovascular events, return to OR and cardiac complications could not be calculated due to zero event counts, and some small-cell results were masked (denoted as “N/A” and “*”, respectively). ICES requires the suppression of all small cells with a count or value of fewer than six (i.e., less than 6) in any publicly released data or publications to protect patient privacy and prevent re-identification.*

**eTable 4.** Outcomes by Volume – sensitivity analysis definition (≥6 PAS hysterectomies performed in the prior year).

| Variable | Total | High | Low | Crude RR (95% CI) | Crude P value | Adjusted RR (95% CI) | Adjusted P value |
| --- | --- | --- | --- | --- | --- | --- | --- |
| Sample Size | N = 748 | N = 227 | N = 521 |  | . |  | . |
|  |  |  |  |  |  |  |  |
| Primary composite outcome | 496 (66.3%) | 122 (53.7%) | 374 (71.8%) | 0.75 (0.66 - 0.85) | <.0001 | 0.70 (0.61 - 0.81) | <.0001 |
|  |  |  |  |  |  |  |  |
| Massive blood transfusion | 484 (64.7%) | 117 (51.5%) | 367 (70.4%) | 0.73 (0.64 - 0.84) | <.0001 | 0.68 (0.58 - 0.79) | <.0001 |
|  |  |  |  |  |  |  |  |
| Any transfusion | 487 (65.1%) | 120 (52.9%) | 367 (70.4%) | 0.75 (0.66 - 0.86) | <.0001 | 0.70 (0.60 - 0.81) | <.0001 |
|  |  |  |  |  |  |  |  |
| Maternal ICU admission | 198 (26.5%) | 25 (11.0%) | 173 (33.2%) | 0.33 (0.22 - 0.49) | <.0001 | 0.40 (0.26 - 0.62) | <.0001 |
|  |  |  |  |  |  |  |  |
| Uterine artery embolization | 54 (7.2%) | 28 (12.3%) | 26 (5.0%) | 0.63 (0.48 - 0.82) | 0.0008 | 0.53 (0.39 - 0.72) | <.0001 |
|  |  |  |  |  |  |  |  |
| Return to ED/Hospital | 162 (21.7%) | 67 (29.5%) | 95 (18.2%) | 1.62 (1.23 - 2.12) | 0.0005 | 1.24 (0.89 - 1.74) | 0.2090 |
| Embolism/Shock/DIC | 39 (5.2%) | 9 (4.0%) | 30 (5.8%) | 0.69 (0.33 - 1.43) | 0.3157 | 1.48 (. - .) | <.0001 |
| Surgical complications | 31 (4.1%) | 7 (3.1%) | 24 (4.6%) | 0.67 (0.29 - 1.53) | 0.3420 | 0.45 (. - .) | <.0001 |
|  |  |  |  |  |  |  |  |
| Return to OR | 22 (2.9%) | *1-5 | *17-21 | * | . | * | . |
|  |  |  |  |  |  |  |  |
| Sepsis | 17 (2.3%) | *1-5 | *12-16 | * | . | * | . |
|  |  |  |  |  |  |  |  |
| Acute renal failure | 7 (0.9%) | *1-5 | *2-6 | * | . | * | . |
|  |  |  |  |  |  |  |  |
| Cardiac complications | 7 (0.9%) | *1-5 | *2-6 | * | . | * | . |
|  |  |  |  |  |  |  |  |
| Death | *1-5 | 0 (0.0%) | *1-5 | N/A | . | N/A | . |
|  |  |  |  |  |  |  |  |
| Cerebrovascular events | 0 (0.0%) | 0 (0.0%) | 0 (0.0%) | N/A | . | N/A | . |
|  |  |  |  |  |  |  |  |
|  | Mean (SD) | | |  |  |  |  |
| Length of hospital stay after index surgery | 4.55 (3.62) | 4.11 (2.64) | 4.74 (3.95) | 0.74 (0.56 - 1.00) | 0.0464 | 0.79 (0.62 - 0.99) | 0.0443 |

*Rare events including surgical complications, acute renal failure, cerebrovascular events, return to OR and cardiac complications could not be calculated due to zero event counts, and some small-cell results were masked (denoted as “N/A” and “*”, respectively). ICES requires the suppression of all small cells with a count or value of fewer than six (i.e., less than 6) in any publicly released data or publications to protect patient privacy and prevent re-identification.*
